# The Impact of Aging on Cardio and Cerebrovascular Diseases

**DOI:** 10.3390/ijms19020481

**Published:** 2018-02-06

**Authors:** Carmine Izzo, Albino Carrizzo, Antonia Alfano, Nicola Virtuoso, Mario Capunzo, Mariaconsiglia Calabrese, Eros De Simone, Sebastiano Sciarretta, Giacomo Frati, Marco Oliveti, Antonio Damato, Mariateresa Ambrosio, Francesco De Caro, Paolo Remondelli, Carmine Vecchione

**Affiliations:** 1 Departement of Medicine and Surgery, University of Salerno, 84081 Salerno, Italy; carmine.izzo93@gmail.com (C.I.); mcapunzo@unisa.it (M.C.); olivetimarco@yahoo.it (M.O.); fdecaro@unisa.it (F.D.C.); premondelli@unisa.it (P.R.); 2 Vascular Physiopathology Unit, IRCCS Neuromed, 86077 Pozzilli, Italy; albino.carrizzo@gmail.com (A.C.); sebastiano.sciarretta@uniroma1.it (S.S.); fraticello@inwind.it (G.F.); antonio.damato85@libero.it (A.D.); mattyambr@gmail.com (M.A.); 3 Heart Department, A.O.U. “San Giovanni di Dio e Ruggi d’Aragona”, 84131 Salerno, Italy; antonia.alfano@sangiovannieruggi.it (A.A.); eros.desimone@sangiovannieruggi.it (E.D.S.); 4 Department of Cardiovascular Medicine, A.O.U. Federico II, 80131 Naples, Italy; n.virtuoso@hotmail.com; 5 Rehabilitation Department, A.O.U. “San Giovanni di Dio e Ruggi d’Aragona”, 84131 Salerno, Italy; mac.calabrese@virgilio.it; 6 Department of Medico-Surgical Sciences and Biotechnologies, Sapienza University of Rome, Polo Pontino, 04100 Latina, Italy

**Keywords:** aging, cardiovascular diseases, genetics, molecular mechanisms

## Abstract

A growing number of evidences report that aging represents the major risk factor for the development of cardio and cerebrovascular diseases. Understanding Aging from a genetic, biochemical and physiological point of view could be helpful to design a better medical approach and to elaborate the best therapeutic strategy to adopt, without neglecting all the risk factors associated with advanced age. Of course, the better way should always be understanding risk-to-benefit ratio, maintenance of independence and reduction of symptoms. Although improvements in treatment of cardiovascular diseases in the elderly population have increased the survival rate, several studies are needed to understand the best management option to improve therapeutic outcomes. The aim of this review is to give a 360° panorama on what goes on in the fragile ecosystem of elderly, why it happens and what we can do, right now, with the tools at our disposal to slow down aging, until new discoveries on aging, cardio and cerebrovascular diseases are at hand.

## 1. Introduction

Over the last century there has been a gradual increase in average life expectancy; in parallel, there has been a rapid increase in diseases associated with aging such as degenerative, cardiovascular and cerebrovascular diseases. The influence of age on cardiovascular and cerebrovascular diseases is due not only to an increase in risk factors in the elderly but also by the independent and inevitable effect of aging itself.

Knowledge of the mechanisms which rule aging in the cardiac and cerebrovascular systems is essential to lay the foundation to change our perspective on the concept of aging no longer as a risk factor that cannot be modified but rather as a modifiable risk factor.

In fact, as a consequence of this perspective a new horizon on how to prevent and how to postpone aging of cardiac and cerebrovascular system con be established, all in order to reduce mobility, disability and health care costs. This research is driven by the idea that the goal is not only to “add years to life but especially to give life to years” and this can only be undertaken by reducing both the physical and cognitive disabilities.

## 2. Structural and Physiological Changes in Heart during Aging

The increase in heart weight is the first event that we can see during aging, with special regard to left ventricular hypertrophy. Interestingly, this phenomenon is present even in healthy subjects with no history of hypertension or other causes that may increase after-load. The increase in myocardial thickness is due to an increase of cardiomyocytes size, whereas the overall number of cardiomyocytes is decreased [1]. Collagen fibres undergo a process of thickening mostly because of an increase in focal deposits and as a consequence to enhanced cross-linking between adjacent fibres [2].

Interestingly, a change in the hearts overall shape can be appreciated, shifting from an elliptical form to a spheroid one, with an asymmetric hypertrophy of the interventricular septum in comparison to the other walls [3]. Moreover, both the change of positioning towards right of the ascending aorta and the protrusion in the interventricular septum is the basis for the reduction of the left ventricular outflow tract [4].

These changes together have an important effect on cardiac wall stress and overall contractile efficiency, above all for diastolic events. In an aging heart at rest, with preserved ejection fraction, stroke volume, heart rate and systolic function seems to be unaffected by the strain of time [5].

The combined effects of the alterations in the shortening velocity and in the heart’s interactions with the vasculature seem to find an equilibrium leaving the systolic function intact at rest. In fact, research on healthy normotensive individuals and their left ventricular (LV) shortening fraction [6] and radionuclide ejection fraction (EF) [7] showed an unaltered cardiac EF at rest (normal average EF > 65%). Of course, this does not mean that the overall components of the cardiac systole are unaltered. On the other hand, significant changes can be seen in the diastolic function in elderly subject, with a para-physiological redistribution of the early diastolic filling volume into an increased end-diastolic filling volume [8].

Other study show that an aging heart undergoes changes in cardiac responsiveness to both pharmacologically and physiologically adrenergic stimuli, in fact heart rate and myocardial contractile ability are unpaired in elderly subjects. As a consequence, the Frank-Starling mechanism is activated by the left ventricle, allowing a marked increase in both end-diastolic and end-systolic volume, in order to compensate cardiac output ensuring the metabolic needs no longer guaranteed by the contractile capacity and chronotropic reserves of the aging heart. This cardiac compensation pattern allows elderly subjects to perform vigorous exercises that may occur in everyday life. Obviously, the increase in maximum cardiac output intensity is not as in the younger individuals, a reduction of 20–30% in peak cardiac output in response to maximal effort can be seen in elderly [9]. In the elderly, the physiological response to exercise is altered by the decrease in myocardial time of relaxation, reduction in responsiveness to β-adrenergic stimuli and in overall pattern of relaxation resulting in a diminished response to cardiac stress in terms of stroke volume and heart rate, losing the “suction” effect present in the younger attributable to the increase in the isovolumetric relaxation due to higher heart rate [3,10,11]. On the other hand, it is important to note that the elderly heart at rest, as a result of the reduction in the heart rate and with a consequent longer diastolic and systolic filling time, is able to maintain both end-diastolic and end-systolic volume (although persisting the reduction in early diastolic filling rate in favour of the later diastolic filling) [12]. Taken together, in the elderly these mechanisms of compensation guarantee an adequate metabolic support at rest while uncovering an insufficient cardiac functional reserve in response to maximal exercise in term adrenergic response, diastolic filling time/rates and not to mention peak-filling rate at increased heart rate.

The heart of an elderly subjects in relation to the above findings seems comparable to a younger heart undergoing a treatment with β-blockers in terms of responsiveness to exercise [13].

Another milestone seems to be the lusitropic function of the aged heart, with the combination of delayed relaxation as both cause and effect of enhanced duration of contraction in relation to prolonged action potentials rather that to mere alterations in mechanical or myocardial properties [14]. The above-mentioned alterations clarify why elderly subjects are likely to develop diastolic heart. In the elderly, the permanence of inotropic responsiveness to calcium ions rather than to digitalis has brought about new clues on the reduction of the signalling process function thus indicating a new path of study rather than focalizing only on the myocardial contractile capacity, however to confirm such hypothesis more studies are needed [15].

As the heart ages the cardiac conduction system undergoes many changes such as progressive fibrosis, accumulation of peri-sinoatrial node fat and calcification on the cardiac skeleton resulting in an increase of resistance in the node area with a reduction of the electric signal propagation. After age 60 the pacemaker cells undergo a pronounced decline in the number, resulting in a loss of 90% of the pacemaker cells present in young adults [16]. The structural degeneration in the sinoatrial node heavily affects the responsiveness to both sympathetic and parasympathetic stimuli of the aging heart, bringing about a reduction of the normal heart rate at rest and a reduction to maximal heart rate during exercise in the elderly [17]. Obviously, the alterations that occur to the sinoatrial node affect the atrioventricular node, atrioventricular bifurcation and proximal left and right bundle branches as well, as a result the risk of atrioventricular conduction block in the elderly greatly increases. The risk of atrioventricular conduction block can be assessed by the P-R interval, which in the elderly settles at a mean of 172 ms at age 60 while from age 20 to 25 it settles at a mean of 159 ms, confirming our concerns [18].

Another important factor is the wall thickening of the LV in the elderly, which brings to a leftward axis shift of the QRS axis [19] and unlike expectations to a decline of the R-wave and S-wave amplitudes, which can be seen already by age 40 [20].

Although further studies are needed to find a clinical heart disease relation between electrocardiography (ECG) and aging, worth noting is the increase in nonspecific ST-T alteration which may shed light on the mechanisms that lay behind the arrhythmias in the elderly [21]. In agreement with previously report, the elderly present an increased susceptibility to supraventricular arrhythmias such as atrial arrhythmias, atrial fibrillation, paroxysmal supraventricular tachycardia and to ventricular arrhythmias mainly extra systole [6].

In fact, Atrial fibrillation (AF) has been found in approximately 3–4% of subjects older than 60, a rate 10 time higher than the general adult population. In 1–2% of healthy individuals older than 65, as shown by twenty-four-hour ambulatory ECG monitoring studies on the elderly at rest, present short bursts of paroxysmal supraventricular tachycardia (PSVT) [22,23].

Furthermore, with advancing age an exponential increase in Ventricular ectopic beats (VEBs) can be highlighted up to an alarming level of 8% in patients above 70 that have been hospitalized [24].

In our clinical practice a significant amount of VEBs related to exercise can be seen in older male adults with no apparent heart disease risk, thus leads us toward considering a correlation between frequent and repetitive exercise-related VEBs and aging as a possible sign of suffering of the cardiac conduction system in the elderly.

Of fundamental clinical importance is keeping in mind and recognizing the ECG abnormalities that may lead to an increase in cardiovascular risk such as increased QRS voltage, Q waves, QT interval prolongation and ST-T-wave abnormalities. In fact, these abnormalities are strongly predictive for left bundle branch block and AF two of the greatest means of cardiac morbidity and mortality in the elderly.

Moreover, another important process worthy of being considered is the aberrant handling of Ca^2+^ and its electro-chemical gradient in the individual cardiomyocyte, which seems to be the main key of arrhythmias. In reference to the aging heart, the reduction in the Ca^2+^ reuptake rate, due to SERCA2 protein levels down regulation and increase in Na/Ca^2+^ ion exchangers, seems to be the trigger leading to transient increase of Ca^2+^ cytosolic levels and to a prolonged action potential and rate of contraction. These changes in ion currents lead to a prolonged systole and diastole bringing about an overall reduction in maximal heart rate in the elderly [25,26].

To prevent however spontaneous Ca^2+^ oscillations and thus arrhythmias, the cardiomyocytes at first implement the L-type Ca^2+^ currents to increase the overall channel number in order to induce a secondary effect of reduction of Ca^2+^ currents by the inactivation of the L-type channel and by the reduction of the inward K1 current. The overall effect is a prolonged action potential which brings to a consequent lusitropic effect due to the increase of the mean time permanence of Ca^2+^ in the cardiomyocyte. However, the permanence of Ca^2+^ inside the cardiomyocytes negatively affect the production of reactive oxygen species (ROS) increasing the lipid peroxidation of polyunsaturated fatty acids (PUFAs) which ultimately react with protein sulfhydryl groups leading to miss folding and altered protein conformation. [25]. In brief, due to alteration of intracellular Ca^2+^ compartmentalization/currents and to excitation/contraction uncoupling the cardiomyocytes in the elderly have a decreased capability in tolerance and adaptation to stress leading to overall reduction of cardiac efficiency resulting in arrhythmias [25,27].

The cardiovascular system, in response to acute stress, activates the adrenergic signalling causing an increase in myocardial contractility and relaxation, increased heart rate, increased blood pressure, reduced LV afterload and vasodilatation in working muscles allowing a greater blood flow.

Aging concerns negatively adrenergic signalling, thus the response of the heart and its components to Adrenalin and Noradrenalin, with a reduction in both postsynaptic b-adrenergic signalling and cardiovascular responses to b-adrenergic antagonist infusion leading to an insufficient increase in heart rate, LV contractility and arterial blood flow [28].

Despite knowledge of several alterations of the adrenergic mechanisms, such as the presence of elevated levels of sympathetic neurotransmitter in response to stress in the elderly [29], the mechanisms remain unclear. Regarding the probable compensatory increase of catecholamine plasma levels in the elderly as a response to functional decline, this is likely a way to make up for a reduction in cardiac muscarinic b-receptor density. As far as the increase of the catecholamine plasma level itself it is maybe due to a set of effect such as reduced plasma clearance, deficient nerve reuptake and increased spill over from tissues [30].

In response to acute stress/exercise the elderly may reach the metabolic need to carry out the job at task, whoever if the exercise is prolonged the abovementioned system of compensation will contribute negatively bringing depletion, reduced release and spill over of the neurotransmitters causing an utterly insufficient cardiac response.

Cardiac remodelling is one of the most interesting arguments in relation to clinical outcome in the elderly. Much is yet to be discovered but many key clues have been identified such as the central role of renin-angiotensin system (RAS), the effect of oxidative stress, the increase in ROS and its feedback and the linking role between these and the nicotinamide adenine dinucleotide phosphate (NADPH) oxidases [31].

Taking a deeper look into the effect of RAS signalling, studies have shown its ability to mimic a growth factor which is activated by the stretch of the cardiomyocytes and fibroblasts in response to an increased load enforced by the stiffened aged vasculature. The observable effect of the RAS signalling coupled with angiotensin II (ANGII) and transforming growth factor beta (TGF-β), as seen in vitro, is hypertrophy of the cardiomyocytes, increase matrix production and interestingly increase in apoptosis [32].

ANGII is released in response to stretch [33] by fibroblast and cardiomyocytes in an autocrine/paracrine manner, binding on its two receptors AT1 and AT2 it performs, directly and indirectly, its role activating many transduction cascades such as the NADPH oxidase complex to generate ROS, of which we will deepen later [34].

## 3. Vascular Alterations during Aging

Just like the heart, with aging the vasculature and its two main components: the vascular endothelium and the media arterial wall undergo several structural and functional changes. Recent studies have underlined that the reduction in vasodilatory capacity of the vascular endothelium is due to the loss of both vasodilator and vasoconstrictor homeostatic factors. The media arterial wall component on the other hand undergoes to a slow decay with increase in calcium deposits, accumulation of extracellular matrix and most of all hypertrophy which, greatly, influences the increase of vascular stiffness in the elderly [35,36].

The most evident structural change is borne by the intima and the media of large arteries which become elongated and tortuous with a thickened wall compensated by an enlargement of the arterial lumen [37].

This reasoning also applies to the Aortic root which undergoes to a process of dilatation and stiffening, as seen in echocardiographic studies in aging subjects [38], leading to an increase in afterload and in heart pump stress in relation to the increased blood volume present in the proximal Aorta. These overall great vessels alteration are the leading cause for LV hypertrophy compensation. Moving deeper into aging vasculature, it is possible to notice that the endothelial dysfunction represents the first step of vascular alterations [16]. In the elderly, we assist to a reduction of the elastin component and to an increase of collagen component, that leads to an increase of media arterial wall stiffness (especially in large vessels) and to a great undermining of the arterial distensibility linked to the elastin ability to transfer the load of blood flow pulse wave throughout the media layers [39]. To guarantee a structural continuity, collagen and elastin are usually kept together by enzymatic cross-linking, however in the aged vasculature there is a shift toward irreversible non-enzymatic glycation-based cross-linking. This mechanism perhaps arises from the need of the vasculature to stabilize the increased fragmentation of the collagen fibres secondary to enhanced matrix metalloproteinase (MMP) activity and from diminished response to mechanical fatigue in the elderly. Obviously, this not only further contributes to arterial stiffness but it also activates an inflammatory stress response to advanced glycation end products which establishes a vicious cycle [40,41].

The keystone of the impaired endothelial vasorelaxation is Nitric oxide (NO). On this regard, several animal studies have shown an aged-related reduction in endothelial nitric oxide synthase activity (eNOS) [42,43], which, leads to a decline in NO production and levels. This theory is confirmed by human studies, in which, it has been demonstrated that in the elderly there is an important reduction of NO bioavailability associated with a marked dysfunction of endothelial-dependent vasodilatation [39]. To make matters worse, there is a frequent coexistence of cardiovascular comorbidities such as hypertension, hypercholesterolemia and atherosclerosis which further negatively affect endothelial dysfunction [44].

As discussed so far, impaired endothelial vasodilatation is always followed by arterial stiffness, thus indicating a connection between endothelial dysfunction and these arterial alteration [45]. Endothelial dysfunction is also correlated with microvascular dysfunction in the aging [46], which seems to be a promoting factor of increased blood pressure, large arterial stiffness and small artery remodelling, all factors leading to multi organ damage in the elderly [47].

A contributing factor to endothelial dysfunction in the elderly is the reduced turnover of the endothelial cells, which are fundamental for operating integrity, maintenance and regeneration. This lack ultimately leads to reduced operating integrity in terms of vasodilatation/contraction and reduced capacity in re-endothelialisation of injured arteries [48,49]. This can be highlighted by the decreased number and malfunction in proliferation and migration of the endothelial progenitor cells (EPCs) in human studies [50]. Furthermore, there seems to be a correlation between aging, large and small artery elasticity and EPCs number and functions suggesting a link between aging, reduced re-endothelialisation and functional and structural changes in aging vasculature [51,52].

Moreover, the age-related structural changes leading to a reduction of the ability of both large and small arteries to enhance lumen promotes an impaired distensibility and increase in the pulse wave velocity and blood pressure [53,54]. The loss of the cushioning function of the Aorta and of the large arteries is due to marked alteration in elastic-type arteries, diminished humoral regulation, reduced nitric oxide-dependent vasodilatation and attenuation of vasodilator responses to B_2_-adrenoceptor agonists [55,56,57]. Surprisingly, healthy aging subject in absence of clinical hypertension are also subject to central arterial stiffening as shown in studies measuring pulse wave velocity (PWV) in the elderly [58]. The main problem concerning the alteration of forward pulse wave is its return, as in young adults it returns in diastole thus assisting coronary artery filling while in the elderly the increase of pulse wave velocity returns in systole causing an increase in ventricle load and reduction in coronary blood flow [59].

Interestingly, similar functional changes and biochemical pathways alterations can be seen in atherosclerosis-free normotensive aging individuals and in atherosclerotic vessels which due to the presence of plaques are just as stiff but have the further disadvantage of being prone to focal lesions leading to vessel stenosis and plaque rupture not to mention chronic inflammation which however can be augmented in normal aging vessels as well. From these assumptions, we can conclude that aging and atherosclerosis are “two sides of the same coin” meaning that aging can be seen as a prodromal stage of atherosclerosis disease and atherosclerosis as a form of accelerated arterial aging.

From an academic point of view, these two processes remain distinct in both origin and progression, as aging does not unavoidably cause focal disease while Atherosclerosis surely will. The objective of future research should however focus on finding and ultimately controlling the genetic and/or the molecular mechanisms that define the switch from benign age-related changes to pathological atherosclerotic degeneration [31,39,60].

Age-related hypertrophy and stiffness, discussed earlier, initiates a negative hemodynamic feedback cycle leading to steady age-related increase in total peripheral resistance, Systolic and pulse pressure.

These adverse phenomena are a strong stimulus of further stiffness and hypertrophy of the media arterial wall, even if its progression can be more or less fast in relation to coexisting comorbidities such as atherosclerosis, dyslipidaemia, smoking and hypertension.

## 4. Decline of Endothelial Vasorelaxation during Aging

Age-dependent endothelial dysfunction is a multifactorial disease associated with vascular endothelial cell deterioration [46]. The main consequence in elderly seems to be a reduction in endothelium-dependent vasodilatations, thus increasing morbidity and mortality [61,62]. Studies, in fact, have shown that maintaining vascular layer health is essential to reduce or at least slow down the pathophysiological mechanisms [61,63], which set aging as an independent risk factor, even in absence of other cardiovascular risk factors [64].

EDHF, alongside nitric oxide (NO) and prostacyclin, is one of the main factors for endothelial vasodilation. EDHFs vasodilatation effect has been studied on the coronary arteries and on other vascular regions in elderly humans [65] and so has been its age-related functional reduction in aged rats. Interestingly, in rats age-related malfunction in EDHF-mediated vasodilation has been prevented by administration of red wine polyphenols which led to a reduction in the expression of calcium-activated potassium channels and to a greater responsiveness of the renin-angiotensin system (RAS) [66,67]. Small-, intermediate and large-conductance calcium-activated potassium channels (SK, IK and BK) seem to play an important role in age-related vascular dysfunction through a decline of the hyperpolarizing responsiveness, as seen in aged rats’ coronary arteries under stress [68,69].

Other molecules, such as cyclooxygenases (COXs), are able to produce contractile/vasodilators factors like TXA2 and prostacyclin and prostaglandin I2 (PGI_2_), respectively [70]. In the elderly, this delicate equilibrium is disrupted, due to lost in prostacyclin endothelium-dependent vasodilatation [71,72] and for increase in COX-derived vasoconstrictor factor, as seen in mesenteric arteries [46], ultimately causing an increase in endothelium-dependent contraction. Much is yet to be discovered on the possible double effect of prostacyclin as both an endothelial vasoconstrictor and vasodilator factor and on the age-related contribute of COX-1 and COX-2 on endothelial-dependent vasoconstriction [73,74,75]. One thing is for sure as research deepens knowledge on this matter and that is that the COX pathway is heavily influenced not only by protein expression but also by the molecular interaction with the NO pathway which augments inflammatory state through increased production of reactive oxygen species [76].

Literature shows how NO is one of the most important molecular factors for cardiovascular health and longevity [77,78,79]. NO is produced by 3 different NO synthase isoforms: neuronal NOS (nNOS or NOS-1), cytokine-inducible NOS (iNOS or NOS-2) and endothelial NOS (eNOS or NOS-3). However, eNOS is the only membrane associated isoenzyme and as such it is involved in regulating vasodilatation in response to sheer stress from increased arterial blood flow. Even if in aged endothelium eNOS expression changes seems to be unclear [43,80,81,82], there is no doubt about eNOS activity reduction [83], leading to a diminished NO regulation. An important Domino effect to keep in mind is the reduction in Nitric oxide bioavailability [84]. In fact, increased pro-inflammatory activity and increased chronic oxidative stress, at the root of NO bioavailability reduction, is a consequence of an overall increase in superoxide production probably due to reduced tetrahydrobiopterin (BH_4_) availability and eNOS uncoupling [85,86,87,88].

Oxidative stress is a pathological condition caused by disequilibrium between reactive oxygen species (ROS) production and elimination. ROS are normally produced in a regulated fashion with a basal rate by oxygen reduction processes mediated in the mitochondrial membrane, biochemical combination of two molecules of superoxide anion (formatting hydrogen peroxide) and by reactions of metal ions and hydrogen peroxide (forming hydroxyl radicals) [89]. ROS physiologically are cell signalling initiators which interfere with post-translational cellular protein folding and redox activation and inhibitor mediated by transcriptional factors [89].

ROS disequilibrium, as a result of both physiological and pathophysiological stimuli, generates accumulation of damaged/misfolded proteins, inflammation, increased mutagenesis and most of all increased endothelial senescence and dysfunction [90].

ROS mediated endothelial senescence and dysfunction seems to be coupled with NO production and signalling and with telomerase alteration both in a direct and indirect way [91]. This may be explained by the close relationship laying behind aging, telomerase shortening and the systems of apoptosis and repair in the endothelial cells [92,93,94,95,96].

Aging vascular walls are susceptible to ROS mediated damage [97]. ROS seems to diminish NO endothelium-dependent relaxation because of superoxide high reaction rate with NO, resulting in a NO activity reduction. Moreover in aging, concentration increase in superoxide levels has been shown to increase reaction rate bringing about a significant biological endothelial damage and dysfunction [98].

ROS reaction with NO generates peroxynitrite (ONOO^−^), which can easily penetrate the cell membrane and due to its high reactivity has been shown to bring to macromolecular, lipid and DNA oxidation associated with aging and premature aging [99,100]. The big problem is however that peroxynitrite (ONOO^−^), a sign of clear NO availability and action reduction, seems to be a ROS production promoting factor, thus establishing a vicious cycle [101].

Many human studies on vessels have underlined an age-related increase in oxidative stress markers and a contemporary reduction in endothelium-dependent dilation in relation to the young [85]. Furthermore, an increase in superoxide production in aging arterial walls and a reduction in NO availability seems to be a determining factor in longevity, meaning that an increase oxidative stress resistance and a decreased vascular ROS production could be the keystone to a healthier and longer life, of course more studies are needed [46].

ROS, NO production and the inflammatory pathway appear to have a linking bond in aging vasculature in both a direct remodelling effect and an indirect long-term impact which can be remarked in increased vascular smooth cell expression of MMP-2 and MMP-9 due to increase of NADPH oxidase and mitochondria ROS production.

The devastating effect of these reactive molecules finds its evidence in improved NO-dependent endothelial vasodilation after superoxide withdrawal [46]. Furthermore, in the elderly oxidative stress withdrawal and SOD enhancement appear to be independent protective factor in subject with or without concurrent cardiovascular risk factors (CVRF) [64].

## 5. Sources of Oxidative Stress and Vascular Aging

The main enzymatic systems of ROS production in human vasculature are NADPH oxidases, uncoupled NO synthase and the mitochondrial respiratory chain [101,102,103].

The superoxide anion (O^−^) is the main free radical produced from electron leaks in the electron transport system in the mitochondria [104]. It is rapidly converted into hydrogen peroxide (H_2_O_2_) through the enzyme superoxide dismutase (SOD1, SOD2, SOD3). The H_2_O_2_ can in turn react with metals in reduced intermediate phase through the Fenton reaction and produce a highly reactive free radical, namely hydroxyl radical (OH^−^) [105,106]. Mitochondrial ROS contribution to the aging process is massive [107,108] and its load can worsen in conditions of inefficient nutrient oxidation causing both arterial stiffness, diminished endothelial vasodilation and increased endothelial apoptosis rate [109]. Based on the mitochondrial theory of aging, ROS produced via mitochondrial respiration attack mitochondrial constituents [110]. In particular, accumulation of oxidant-induced somatic mutations in mitochondrial DNA (mtDNA) is believed to be the underlying cause of the decline in physiological function with age. Since the heart is an organ with high aerobic metabolism, the role of mitochondria is most important. In fact, cardiac tissue is more sensible to oxidative damage [111]. On this regard, some evidences revealed that cardiac mitochondrial DNA undergo to major damage during aging since cardiomyocites have a reduced ability to produce antioxidant defence compared to other cell types. The best evidence that mitochondrial ROS exert a fundamental role in cardiac protection has been demonstrated in mCAT (mitochondria catalase) transgenic mice, showing that these mice had 18% of lifespan prolongation compared to their littermates [112].

Furthermore, mitochondrial ROS in its cross-linking with the inflammatory pathway exerts a lead role in activating transcription factors, like NF-κB and AP-1 [113]. Recent studies have demonstrated that mitochondria-derived ROS, produced during inflammatory status, lead to a severe vascular dysfunction. This effects seems to be mediated by a downregulation of Nrf2 pathway thus promoting a reduction of antioxidant defence against oxidative stress [114], reducing nitric oxide bioavailability, increasing production of vasoconstrictors like endothelin-1 and activating many proinflammatory genes [115]. Based on these data, it is clear that mitochondrial ROS signalling is implicated in the regulation of vascular tone and that its excessive production leads to a disruption of normal ROS signalling and mitochondrial dysfunction, which contribute to the pathogenesis of vascular disease.

ROS generated from NADPH oxidases (NOX) plays an important role in age-related free radical production [116]. There are seven NOX types that oxidize reduced coenzyme NADPH producing the oxidized form NADP^+^ and H_2_O_2_.

These transmembrane enzymes deliver electrons to oxygen NADPH one at a time, through a flavoprotein and cytochrome associated with it, so the initial product of the reaction is the univalent derivation of the oxygen reduction of, the superoxide anion, O^−^. The superoxide can then reach the cytoplasm through anion channels or be quickly turned into hydrogen peroxide diffusing through membranes [117,118,119,120,121,122,123].

Age-related NADPH oxidase-derived ROS endothelial dysfunction is suggested by new findings on human mesenteric micro-vessels in which increased expression of the NOX-4 subunit of NADPH oxidase and improved endothelial vasodilation after NADPH oxidase inhibition with apocynin was proven [46].

The NOS uncoupling phenomenon is the consequence of NOS activation in absence of either l-arginine or BH_4_, eNOS causing superoxide production [77,124]. eNOS seems to play an important role in aging process due to increased ROS production [125].

In fact, supplementation of BH_4_ with a correspondent enhancement of the intracellular levels of BH_4_ has been shown to prevent NOS uncoupling and improve endothelial function in healthy elder subjects [46]. Same is true in healthy elder subjects treated with oral administration of l-arginine [126]. Arginase also seems to play a decisive role in reduction of NO synthesis by degrading l-arginine, thus decreasing substrate availability for eNOS [127]. The logical consequence of this argument was supported by improved endothelial function and arterial compliance in aging vasculature secondly to upregulation of arginase [128].

Interestingly, both eNOS and iNOS are subject to uncoupling in absence of the same substrates [129].

Different ROS sources appear to be working in synergy in contributing to vascular aging. Mitochondrial ROS, activation of NADPH oxidases and uncoupling of NOS establish between one another a positive feedback causing an overall increased concentration of ROS [130]. This intricate system of cross-talk can be seen as a feed-forward cycle where mitochondrial ATP-sensitive potassium channels (mitoK_ATP_) are activated by superoxide and H_2_O_2_ [131,132], which in combination with NADPH oxidase activity causes an increase in angiotensin II (AngII) mediated mitochondrial ROS production [133], which to close the feed-forward cycle enhances mitoK_ATP_ [133].

Chronic low grade inflammation in aging seems to be the main factor contributing to increased basal ROS rate production [44]. The combination in the elderly of ROS production increase and Chronic low grade inflammation is featured by an enhanced boost plasmatic concentration of inflammatory markers among which the most important are tumour necrosis factor alpha (TNF-α), interleukin 1beta (IL-1β), members of the super family of interleukin 6 (IL-6), as well as higher levels of C-reactive protein (CRP) [134]. Interestingly, age-related inflammatory markers increase seem to be an independent CV risk factor, accelerating arterial wall changes in terms of both stiffness and endothelial dysfunction [135,136,137].

Focusing on age-associated vascular inflammation, one of the main mechanisms is represented by NF-κB activation that leads to increased levels and activity of inflammatory factors such as TNF-α, Interleukin (IL-1β, IL-2 and IL-6), chemokines (IL-8 and RANTES), adhesion molecules (ICAM and VCAM) and enzymes (iNOS and COX-2) [138].

Starting from the top, age-related increase in IL-6 has been associated with both vascular dysfunction and increased disability and mortality in the elderly, suggesting a direct correlation between mortality in the elderly and IL-6 inflammatory mediate vascular dysfunction [139]. These factors have also been directly correlated to age related arterial stiffening, due to CRP increase and to arterial intima thickening, due to MCP-1 and matrix metalloproteinase overexpression [140,141,142].

Arterial wall aging is also accelerated by up-regulation of TNF-α, firstly for a direct effect as seen on human coronary arteries [143] and secondly by an indirect effect due to its pro-inflammatory and pro-endothelial dysfunction ability [144]. NADPH oxidase activation [145], endothelial apoptosis [143], NOS uncoupling increase (upregulation of iNOS mRNA expression) [146], reduction of endothelium-dependent dilations (down regulation of prostacyclin-mediated vasodilator and up regulation of thromboxane A_2_) [46] have all been documented in aging human vessels as a consequence to exogenous TNF-α administration.

A feed-forward cycle can be highlighted between ROS activation and the inflammatory state in the elderly. The connecting link can be found in the ability of pro-inflammatory cytokines and in general of local inflammation to upregulate MMPs activity in aging vessels contributing to ROS production [147,148,149].

The NF-κB (“nuclear factor kappa-light-chain-enhancer of activated B cells”) is a protein complex functioning as a transcription factor. NF-κB can be found in all types of cells and is involved in all the cells reactions to stimuli such as stress, cytokines, free radicals.

NF-κB is the heterodimer formed by Rel proteins and p50 [150]. When it is in an inactivated state, NF-κB is located in the cytosol, bound to an inhibitory protein IKB (such as IKBα) [128]. Through the intermediation of the integral membrane receptors, a variety of extracellular signals can activate the enzyme IKB kinase (IKK) [137,151]. The IKK in turn phosphorylates IKBα protein leading to its ubiquitination and degradation in the proteasome. In this way, NF-κB is made available.

The activated NF-κB is subsequently translocated into the nucleus where it binds to specific DNA sequences known as response elements (RE). The complex DNA/NF-κB then invokes other proteins such as coactivators and that the RNA polymerase transcribes the DNA into mRNA, which, finally, is exported to the cytosol and translated into protein. This leads to a change in the functions of the cell, such as the production of pro-inflammatory cytokines, proliferation, apoptosis and cellular aging [152]. NF-κB also activates transcription of mRNA coding for its inhibitory subunit IKB, thereby generating a negative feed-back circuit.

By virtue of its ubiquity, increased activation of NF-κB was easily reported in human vessel aging, highlighting its positive role in inducing gene expression leading to oxidative stress [146,153]. NF-κB expression in endothelial cells undergoes an independent age-related increase developing endothelial dysfunction [46,85].

The NF-κB factor is activated by TNF and by all its stimulating factors, seen earlier [154]. Particular significance, has been attributed to over production of Mitochondrial ROS in vascular endothelial and smooth muscle cells due to the ability of its by-products, superoxide and H_2_O_2_, to increase the gene expression of cytokines (TNF-α, IL-1 and IL-6), adhesion molecules (ICAM, VCAM) and pro-inflammatory enzymes (iNOS, COX-2) resulting in accelerated senescence and endothelial dysfunction [154,155]. At support, Evidence show an improved brachial artery flow-mediated dilation in older humans in response to an increased NF-κB inhibitor expression [156].

The renin-angiotensin system (RAS) is a hormonal mechanism that regulates blood pressure, the circulating plasma volume (blood volume) and the tone of the arterial musculature through different mechanisms.

The primary control centre of the renin-angiotensin system is found in the juxtaglomerular apparatus, where renin is produced and stored. Its biological effect acts on a plasma protein synthesized by the liver, called angiotensinogen, transforming it into the decapeptide angiotensin I. This blood protein is then transformed by a converting enzyme (called ACE, the Angiotensin Converting Enzyme) in an octapeptide angiotensin II, the main bioactive product.

Angiotensin II (which is the most potent vasoconstrictor of our body) is responsible for the aforementioned biological effects of the renin-angiotensin system, that is performed through interaction with specific receptors (AT1 and AT2). Furthermore, Angiotensin II seems to have an endocrine, both autocrine/paracrine, hormonal effect [157].

In the elderly, RAS signalling cascade factors in the arterial walls are strongly increased, above all local ANGII concentration (1000 times higher than plasmatic level). Research shows how ANGII is central in cellular and molecular arterial aging pathway [158,159].

## 6. Age-Related Cerebrovascular System Modifications

Cerebral autoregulation is a process which aims to maintain an adequate and stable blood flow in the brain. While most of the body’s systems show a certain degree of self-regulation, the brain is very sensitive to hyper- and hypo-perfusion, so cerebral autoregulation plays an important role in maintaining adequate blood flow to that organ. The perfusion of the brain is essential for life since the brain has a high metabolic demand, providing enough blood containing oxygen and nutrients to the brain tissue. With aging cerebral autoregulation becomes impaired [160]. Response to blood pressure, O_2_ tension, CO_2_ tension and cerebral metabolism in diminished due to microvascular alterations and due to [160] uncontrolled hypertension [161], cardiac dysfunction [162] and smoking. These conditions and aging may have a devastating impact on the cerebrovascular system.

The blood-brain barrier (BBB) is an anatomic-functional unit that has the primary protective function, acting as a filter for the brains bloodstream. During aging, the ability of the BBB to selectively uptake and transport nutrients and hormones through specific carriers is reduced as seen in aged animals [163]. Moreover, age-related arterial wall endothelial alterations, such as thickening, distensibility reduction, apoptosis and glial proliferation as an inflammatory process, may play an important role in BBB permeability dysfunction, leading to multi-infarct dementia (MID) and neurodegenerative disease [164,165,166]. Essentially, BBB alteration in aging exposes brain to altered metabolism and increased toxin exposure increasing brain cell vulnerability to acute insults such as strokes.

The physiological mechanisms underlying vascular autoregulation of cerebral blood flow are varied and aim at modulating the arteriolar tone to maintain a constant flow during variations of cerebral perfusion pressure. In aging, reduced cerebral blood flow (CBF) is a determining factor for CVD [167]. Moreover, many factors contribute, such as atherosclerosis, auto-regulatory dysfunction and cardiac dysfunction [168]. Cerebral blood flow (CBF) reduction is age-proportioned and seem to effect grey matter more than white, due to its higher metabolic rate [160,169]. A slight decrease in age-related brain metabolic however does not balance supply-demand due to CBF reduction [167].

CBF dysfunction has been associated with MID to Alzheimer’s disease [170,171].

A topic for further vascular dementia research includes labile blood pressure, which the elderly frequently has because of autonomic dysfunction, medication side effects and heart disorders [167]). Further studies with blood viscosity, aging and dementia remain to be conducted [168].

The vascular autoregulation responsible for adjustments depends on the integrity of the endothelium. With age this autoregulation is gradually decreasing because of degenerative alterations of the vessel wall (atherosclerosis). If in fact the autoregulation is made difficult by the presence of arterial atherosclerotic lesions, arterial stiffness and overall dysfunction. There is greater risk that the blood flow decreases to “anoxic” levels for the non-adequate priming of changes in arteriolar tone. Anaerobic glycolysis leads to lactate accumulation, decreasing pH levels in the brain, causing an acidotic state altering the excitability of neurons leading to a loss of autoregulation [172]. Changes in the brain metabolism may generate free radicals and promote influx of calcium and sodium into cells furthering cell injury and death. Free radicals may damage the phospholipids of the cell membrane.

In conclusion, aging appears to affect resistance to ischemia and brain metabolism but more studies are needed to confirm such hypothesis.

## 7. Brain Stroke

Stroke is a vascular disease that affects brain function and produces a large number of symptoms. It is related to an interruption of the blood flow to the brain tissue, due to the closing or breaking of cerebral artery. In the elderly population, the incidence of stroke is between 20% and 35% but stroke is not just a disease of the elderly [173].

In the Ischemic Stroke, due to the closure of a cerebral artery, the brain cells that were previously fed by that specific artery suffer from the lack of nutrients that occurs in the infarcted area and undergo death. Cerebral ischemia represents 85% of all cases of cerebral stroke. An artery may quit because inside a clot (“thrombus”) is formed and goes to permanently close an irregularity of the artery wall itself (the atheromatous plaque): also, known as cerebral thrombosis; or because the artery is reached by parties of clots from afar (“emboli”); usually emboli come from the heart or from atherosclerotic plaques of the arteries that carry blood to the brain: in the latter case, we speak of cerebral embolism.

While, the haemorrhagic Stroke is due to rupture of a cerebral artery, for this reason it is known also as cerebral haemorrhage. It represents 15% of cases of cerebral stroke. The most frequent cause is hypertension, which determines the breaking of normal or malformed vessels.

Worldwide, every year, 15 million people are affected by stroke, nearly 6 million die. Throughout the Western world, stroke is the cause of 10–12% of all deaths per year, it is also the leading cause of disability and the second leading cause of dementia and loss of independence. Because of its high incidence, cerebral stroke is a welfare problem and social rehabilitation is an issue of enormous proportion [174]. It is well-known that some intrinsic risk factors cannot be changed: for example, age, sex, family history. Others can be modified, among them most important are: hypertension, heart disease, diabetes, transient ischemic attack (TIA), smoking and obesity. Moreover, as mentioned before, another important risk factor not to be underestimated, is the atrial fibrillation, abnormal heart rhythm that causes more than 20% of stroke. On this regard, it has been demonstrated that a healthy lifestyle and a proper diet reduces the risk of stroke and help the medications to control the pressure, cholesterol and blood sugar levels. If the risk of stroke is related to the presence of an atherosclerotic plaque blocking the carotid artery, the surgical removal of plaque may significantly reduce the risk of a new, more serious stroke in most cases [175].

In addition to the therapies used in an emergency, in addition to physiotherapy, there are several drugs given to prevent stroke or its recurrence. The drugs used for the Stroke prevention are of different types. For example, earlier antiplatelet therapy, anticoagulant therapy and reasonable antiarrhythmic therapy are the milestone of pharmacologic intervention. Moreover, as has been shown, 3 out of 4 strokes could be avoided by simple accurate early diagnosis and treatment of AF [176,177].

Cerebral haemorrhage consists of bleeding within the brain with blood pouring into the tissues that compose it. It is caused by the rupture of a cerebral blood vessel, typically an artery, as a result of both physical trauma or non-traumatic injuries. Cerebral haemorrhage can, for example, be caused by severe head trauma or conditions, such as emboli or congenital malformations, which undermine the resistance of cerebral vessels facilitating breakage. Even therapy with anticoagulant drugs, as well as the coagulopathies and hypertension, may increase the risk of cerebral haemorrhage [178,179,180].

As expected, symptoms may vary depending on the haemorrhagic location and may appears both suddenly or after some time from acute event. In addition, the onset of these symptoms may progressively worsen or develop very quickly. As has been reported, this condition is life-threatening and is a medical emergency: the accumulation of blood inside the skull can compress the delicate brain tissue, restrict blood supply and bring a sudden increase in intracranial pressure, which can lead loss of consciousness, coma or death. Some patients may even go into a coma before there are any signs of neurologic alterations [181,182].

**Figure 1:**
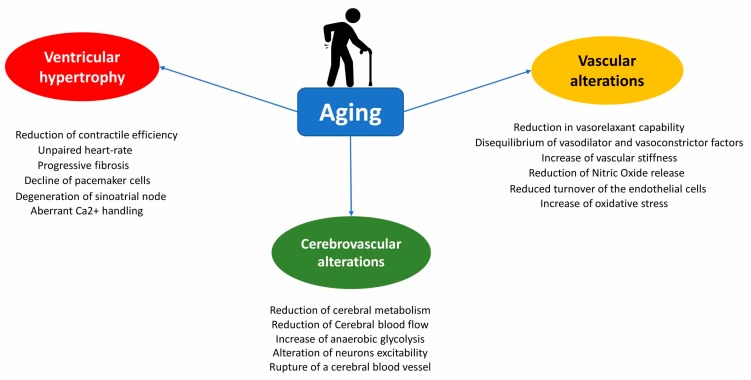
Schematic representation of the major cardio- and cerebrovascular processes involved in the aging progression.

In Elderly, haemorrhagic events incidence is increased because of various vascular structural alterations (aneurism, arteriovenous malformation), hypertension, amyloid deposits, increased cerebral traumatic rate and lastly AF, which also contributes to both stroke and haemorrhagic conversion of their stroke. It is well-known that the elderly ongoing to the vascular neurocognitive disorder (vascular dementia), a form of cognitive impairment and the alteration of the cerebral blood circulation resulting from acute events, such as a stroke or cerebral haemorrhage, or in chronic vascular diseases, such as atherosclerosis. As in other types of dementia, also in this case the deterioration of specific intellectual abilities depends on area were the degeneration of nerve cells in the brain takes place, determining factor for neuronal damage [183]. In addition, in patients aged over 60, the risk of experiencing stroke or chronic cerebrovascular disease and secondarily develop of vascular dementia are increased by the presence of several cardiovascular diseases such as diabetes, hypertension, dyslipidaemias and heart disease (history of heart attack infarction and atrial fibrillation) [184,185,186,187,188] (Figure 1).

## 8. Genetic Impact on Aging

Based on different model organisms, such as yeast Saccharomyces cerevisiae, the nematode *Caenorhabditis elegans* and the *Drosophila melanogaster* have been identified several genes associated with longevity that seem to exert a protective action against the incidence of cardio- and cerebrovascular diseases. For this reason, several studies have focused the attention on the characterization of the pathways recruited by these genes and its possible role in the regulation of longevity in animal models. In humans, the exceptional longevity is a complex trait. Long-living individuals have delayed aging and a low incidence of vascular diseases. Thus, the aim of the scientific community in the last years was been to evaluate the possible role of the candidate-genes in cardio and cerebrovascular diseases in human.

### 8.1. Sirtuins

The well-known evolutionary conserved enzymes, that act as deacetylases and ribosyltransferases, are represented by Sirtuins. Based on the different intracellular localizations it is possible distinguish in mammalian 7 sirtuins, which participate to several cell functions such as DNA damage repair, cell cycle, metabolic response to nutrient availability and protection from neurological degeneration [189,190,191]. SIRT1 is predominantly nuclear protein but it has been demonstrated that it is able to translocate rapidly from nucleus to cytoplasm, depending on the cellular type and energy status of cell [190]. Recently, several studies have observed that SIRT1 is able to regulate the AMPK pathway through deacetylation of the liver kinase B1 (LKB1) [192]. Moreover, SIRT1 regulates the Insulin/IGF-1 pathway through modulation of UCP2 expression and direct regulation of the IGF-1 signalling pathway [193,194]. Regarding the progress and development of cardiovascular disease, mice lacking SIRT1 gene show greater injury following ischemia-reperfusion process and this injury is reduced in SIRT1 transgenic mice [195].

Interestingly, non-physiological overexpression of SIRT1 (20 fold) in cardiomyocytes leading to oxidative stress and apoptosis, while controlled overexpression of SIRT1 is able to delay cardiomyopathies due to advanced age [196,197].

Several studies have investigated the possible role of SIRT1 in the modulation of blood pressure and cardiac hypertrophy. On this regard, it has been demonstrated that SIRT1 regulates blood vessel growth through a Notch signalling and inhibits angiotensin II-induced smooth muscle cell hypertrophy [198,199]. Arterial stiffness, another important cardiovascular risk factor is regulated by SIRT1 preventing hyperphosphatemia-induced arterial calcification [200].

Between other sirtuins, SIRT2 and SIRT3 seem to be involved in the cerebrovascular homeostasis. In fact, it has demonstrated that these two sirtuins exert a neuroprotective effects during cerebral ischemia preventing neuronal death due to excitotoxicity [190]. In addition, it has been reported that in SIRT3 knockout mice, there are age dependent hypertension and cardiac hypertrophy [201].

SIRT6 has been mainly characterized for its role in the cellular response to inflammation. Recently, other studies have reported the ability of SIRT6 to control cellular senescence and aging though the modulation of oxidative stress [202,203].

In a recent study, SIRT7 knockout mice develop cardiac hypertrophy and inflammatory cardiomyopathy and are also characterized by an increase in fibrosis [204]. In detail, it has been demonstrated that cardiomyocytes from SIRT7 knockout mice have decreased resistance to oxidative stress and an increase in apoptosis.

Even though several beneficial functions of Sirtuins on cardiovascular system has been described, the molecular mechanisms by which they exert the positive effect on CVD remain unknown, so many questions remain to be answered regarding the protective levels of sirtuins.

### 8.2. Insulin-Like Growth Factors

The insulin-like growth factors (IGFs) are involved in the development and function of almost all organs of the body [205]. IGF-1 was one of the initial genes to be identified as a longevity gene, since the lifespan of *C. elegans* was greater when it lost *IGF-1* [206] but in contrast, *IGF-1* knockout mice die shortly after birth [207]. Recently, it has been demonstrated that IGF-1 is able to act on the survival and cells proliferation in vitro [208] and its action is mainly mediated by AKT, which has a pivotal role in preventing programmed cell death by acting on the activity of several proteins involved in the apoptotic cascade [209]. The IGF-1 effect is mediated by the binding with the specific receptor IGF1-R. In fact, IGF-1 receptor null mice die postnatally due to respiratory failure [210]. To this regard, it has been demonstrated that IGF1-R is a central regulator of mammalian lifespan, since the IGF1-R heterozygous mice display greater resistance to oxidative stress, an important mechanism leading to aging progression [211].

In the heart, the behaviour of IGF-1 is contrasting. In fact, in some experiments, the overexpression of IGF-1 reduces cardiomyocytes death and prevents hypertrophy after acute myocardial infarction [212]. In another experimental series, long term exposure of cardiac tissue to IGF-1, leads to heart failure after cardiac ischemia/reperfusion [213,214]. Based on these data, IGF-1 exerts different effects based on the time of exposure and on the paracrine effects of IGF-1.

Despite the well know effects evoked by IGF-1 on the cardiovascular health, more studies are needed to characterize the mechanisms recruited by IGF-1 and to clarify its different tissue-specific actions.

### 8.3. Forkhead Box Proteins

FOX (Forkhead box) proteins are a family of transcription factors that play important roles in regulating the expression of genes involved in several cells processes. FoxO transcription factors are present in all eukaryotes. In mammals, there are four FoxO isoforms, including FoxO1, FoxO3A, FoxO4 and FoxO6 [215], that play a prominent role in regulating expression of genes involved in cell growth, differentiation, proliferation and longevity [216].

Multiple stresses are able to promote nuclear translocation and activation of FoxO transcription factor, which directs the transcriptional program controlling metabolism, longevity and stress resistance. In cardiovascular field, it has been reported that the absence of FoxO1 promotes a cardiac alteration and an impairment of vessels growth [217]. In a mouse model of myocardial infarction, it has been demonstrated that upregulation of FoxO prevents cellular injury by activating pro-survival factors, reducing heart size and incidence of heart failure [195]. The main FoxO isoforms in human endothelial cells are represented by FoxO 1 and FoxO 3a [218]. Both proteins are involved in the regulation of eNOS expression. In fact, FoxO 1 or FoxO 3a deficiency led to a downregulation of eNOS expression reducing new vessels formation and angiogenesis [218,219].

Although the role of FoxOs transcription factors in the cardiovascular system has been extensively demonstrated, several studies are needed to understand the mechanisms through which FoxO s modulate the cardiovascular function and the aging progression in different cell types.

### 8.4. Clock 1

Clock-1 (CLK1/MCLK1) is a mitochondrial enzyme necessary for the biosynthesis of ubiquinone (coenzyme Q), the essential electron transporter of the mitochondrial respiratory chain [220,221]. It has been demonstrated that Clock-1 functional alteration leads to a deregulation of several physiological such as cell cycle, embryogenesis, post-embryonic development and significantly reduces life span in *C. elegans* [222]. Interestingly, the phenotype of clk-1 mutants has attracted particular attention due to its ability to increased life span [222]. On this regard, it has been shown that MLCK1 knockout, leads to embryonic lethality in mice [223]. In addition, it has been demonstrated that MLCK1 heterozygous animals have an increased lifespan, suggesting that MCLK1 levels exert an important anti-aging effect. Moreover, although MCLK1 heterozygous mice have increased mitochondrial oxidative stress, this is accompanied by decreased oxidative damage of cytoplasmic proteins. These finding provide a genetic model that will help to clarify in the future the links between mitochondrial function and aging. 

The effects of MCLK-1 have assessed just on cardiovascular system function and disease progression. MCLK1^+/−^ mutant mice have an enhanced resistance to cerebral ischemia/reperfusion at all ages as showed by a low level of oxidative stress and cells damage [224]. Although the physiological and molecular consequences of aging have been studied in detail, it has been difficult to establish a direct causal relationship to life-span and MCLK1.

### 8.5. p66shc

The mammalian Shc locus encodes for three different isoforms of proteins carrying a Src-homology 2 domain that differ in their N-terminal region. p66shc has a unique N-terminal region which gives it the function of a redox enzyme that has been implicated, respectively, in the cytoplasmic propagation of growth and apoptogenic signals [225]. Accumulation of oxidative cellular damage, is actually considered one of the major cause of ageing progression. On this regard, it has been demonstrated that the mouse *p66shc* gene induces stress resistance and prolongs life span [226].

About the cardiovascular system, it has been demonstrated that the loss of p66shc inhibits the degeneration of cardiac progenitor cells, protecting from apoptosis and necrosis thus reducing heart failure damage [227]. Moreover, the ablation of p66shc protects from Angiotensin II–induced hypertrophy and cellular death [228]. Also in the pathogenesis of atherosclerosis, loss of p66shc protects vessels from oxidative stress damage [229].

Based on the results until obtained, genetic and biochemical investigation of the p66shc pathway should lead to better understanding of the control mechanisms and relationships between ROS metabolism, cellular survival and ageing process.

### 8.6. Klotho Gene

The *Klotho* gene, localized on chromosome 13 in human, since a long time is an anti-aging gene because it has been demonstrated that mice deficient of *Klotho* show a premature phenotype and a reduced lifespan, while overexpression of Klotho increases lifespan [230].

*Klotho* encodes a single-pass transmembrane protein composed by extracellular domain, a single transmembrane domain and an intracellular domain. Until now, the main identified action of membrane Klotho is about its function as co-receptor of fibroblast growth factor 23 (FGF23) to regulate phosphate homeostasis [231,232]. Cheng L. et al. have demonstrated that PPARγ regulates vascular smooth muscle cell calcification through the activation of Klotho [233]. While, another research team has demonstrated that the downregulation of Klotho is also involved in the modulation of microalbuminuria in subjects with type 1 diabetes [234]. Recently, it has been reported that Klotho proteins are expressed in human cardiomyocytes and in the sinoatrial node region, its level are down-regulated in higher cardiovascular risk patients [235] and in the alteration of sinoatrial node to function under stress [236]. Based on the data reported until now about the Klotho actions, it is clear that several studies are needed to characterize the biochemical relationship between Klotho and lifespan.

### 8.7. BubR1

The protein kinase BubR1 is a central component of the mitotic spindle assembly checkpoint (SAC) [237]. About ten years ago it has been demonstrated that progressive reduction of BubR1 expression in mouse embryonic fibroblasts leading to cellular senescence [238]. Moreover, Bub3/Rae1 haplo-insufficient mice develop early aging-associated phenotypes in which the age runs much faster than normal mice [239]. Investigating the vascular phenotype of mutant mice with low levels of BubR1, mice result in phenotypic changes reminiscent of vascular aging in humans and suggest a role for BubR1 in suppressing the vascular aging process [240,241]. Moreover, aging-related deficiency of BubR1 and subsequent inability to modulate the reactivity to ROS lead to reduced proliferative capacity of aged smooth muscle cells [242]. Interestingly, arterial wall thickness were reduced in loss-of-BubR1 mice, with increased fibrosis and reduced elastic properties favouring the development of cardiovascular diseases [240]. Recently, it has been demonstrated an important role of BubR1 in the regulation of neuronal maturation, showing that during age-related cognitive disability the level of this mitotic checkpoint kinase, decreases with natural aging inducing elderly features and alteration of central nervous system [243].

Based on these results, BubR1 represents a gene with a huge potential to be investigated to discover new possible therapeutic strategies to prevent cellular aging and cardio- and cerebrovascular alterations.

### 8.8. Bactericidal/Permeability-Increasing Family B Member 4

Recent studies have shown that the rs2070325 mutation in the gene *Bactericidal/Permeability-increasing Family B member 4* (*BPIFB4*) is enriched in long-lived subjects of three independent populations, recruited for the Southern Italian Centenarian Study [244], the German Longevity Study [245] and the New England Centenarian Study [246]. BPIFB4 is an *N*-glycosylated protein of 64 KDa belonging to Bactericidal Family Permeability Increasing Protein (BPI)/lipopolysaccharide binding protein (LPB)/palate, lung and nasal epithelium clone (PLUNC) that also includes cholesteryl ester Transfer Protein (CETP) and Phospholipid-transfer protein (PLTP). Expression analysis, have identified the transcript corresponding to BPIFB4 in embryonic stem cells from mice, in the salivary glands, in the testis, in the spleen, foetal heart, into the fabric bone, liver, pharynx mouse, in EPCs (endothelial progenitor cells), in iPS (induced pluripotent stem cells generated from human fibroblasts) in the mononuclear cells and in endothelial and smooth muscle cells.

Until now, the specific role of the protein was unknown. Recently our group has demonstrated the ability of the variant BPIFB4 associated with longevity, to induce an improvement in vascular reactivity in opposition to changes in age, restoring the endothelial function in older animals, by acting on the activation of PKCα and eNOS [247,248]. Moreover, a recent publication has demonstrated that the serum level of BPIFB4 are closely associated with health status of long living individuals [249] corroborating its possible involvement in the anti-aging mechanisms.

Although are necessary many studies aimed at characterizing the molecular effects of the longevity associated variant (LAV) of *BPIFB4*, the data collected, allow us to speculate on the possible therapeutic use of LAV-BPIFB4 in the presence of eNOS dysfunctions that occur during the natural aging process.

### 8.9. The Role of Telomeres in the Aging

Telomeres are specialized DNA-protein complexes located at both ends of eukaryotic chromosomes [250]. Their major role is to prevent the recognition of chromosomal ends as double-stranded DNA breaks, thus preserving genome integrity and stability. More evidences have demonstrated that telomeres are highly sensitive to oxidative stress which rapidly accelerates the cellular aging promoting the increase risk of cardiovascular diseases development [251]. In fact, it has been demonstrated that the shorter telomere length was associated with hypertension, increased insulin resistance and oxidative stress [251]. Moreover, another important parameter linked to telomeres shortening is represented by cell senescence. Senescent-positive endothelial cells have been found in atherosclerotic plaque and clearly cell senescence induced by telomere shortening contributes to the development of and progression of atherosclerosis [252]. Patients with chronic heart failure (CHF) have 40% reduced telomeres compared with age- and gender-matched controls and the degree of telomere shortening is associated with the severity of disease. The same condition of telomeres has been reported for the cerebrovascular diseases such as stroke and vascular dementia, in which seems to be confirmed that the individual antioxidant capacity is an important factor in governing telomere shortening rate, the adult telomere length and the progression state of the disease [252]. If the accelerated telomere shortening is cause or consequence of cardio and cerebrovascular aging diseases remains still unknown.

## 9. Conclusions

There is a large amount of evidence indicating that aging is the true new disease of the 21st century that plays a crucial role in the development of neurodegenerative, cerebrovascular and cardiovascular disorders. In particular, as described above, vascular aging encompasses such a wide and complex range of phenomena both at functional and molecular levels that have been studied mainly as separate branches in research, for this reason it is clear that the integration of these branches of research will lead to a better understanding of the functioning of several physiological and pathophysiological mechanisms in the elderly population and will help to improve the therapeutic approaches and consequently the patients’ outcome.

Although still at an early stage, stem cells may be considered a fascinating new approach for replacing the elderly damaged tissue. Research has shown the existence of resident cardiac stem cells and their many homeostatic and repairing abilities [253]. These cells, like all the rest, undergo aging and as such they lose their self-renewing ability ultimately leading to overall cardiac aging and as mention before to a reduction of cardiac function [254,255]. Obviously, an intervention on these cells such as their replacement/transplantation or enhancement through factor-based therapies could be an innovative way to reduce overall cardiac aging. This reasoning can also be applied to endothelial cells as research has shown the existence of endothelial progenitor cells which also have repairing abilities and which health is associated with endothelial aging [254,256]. Acknowledged the great potential of this new therapeutic frontier, we must also acknowledge that a greater understanding of the biology behind stem cells, progenitor cells and behind the mechanisms that regulate aging and rejuvenation are required.

Another interesting therapeutic option is antioxidant supplementation, of course this choice is led by the logic assumption that an increase in antioxidant factors would reduce all the damages that not only leads to aging but also accelerates the aging of the cardiovascular and cerebrovascular system [257,258]. Among the main antioxidants used for therapeutic purpose we must mention: vitamin C that acts through the inhibition of lipid peroxidation and through the regeneration of vitamin E in cell membrane; vitamin E which on the other hand acts directly on oxygen radicals eradicating their damaging effects; Carotenoids that act mainly by quenching singlet oxygen; and Resveratrol which increases endothelial NO production. Although these antioxidants have been shown to reduce levels of isoproterenol in both animal and human but there is still no current evidence that recommends these supplements as a mean to prevent oxidation related aging [259]. This is mainly due to the lack in knowledge in the pathways and the pathogenesis that lead to the aging process and to the lack in relationship between knowledge of the different antioxidant factors, their effects and a reliable marker to assess their potential benefits.

**Figure 2:**
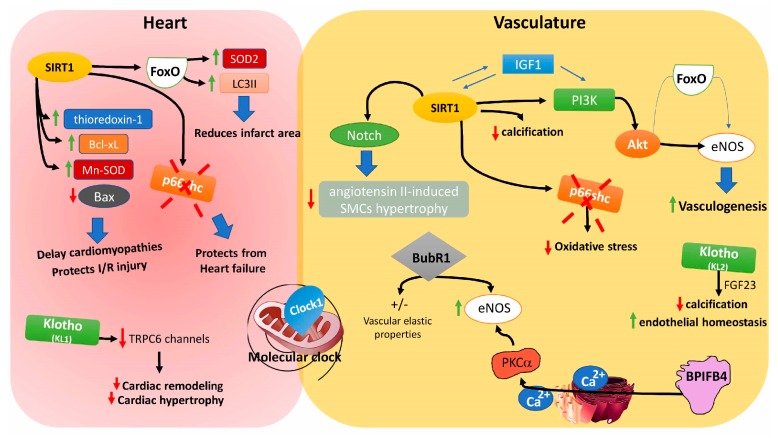
Schematic of the main genes involved in aging regulation and their related molecular mechanisms in heart and vasculature. The green arrows indicate the increase in production/expression; the red arrows indicate the decrease in production/expression.

Furthermore, although several genes involved in regulating the aging process have been identified (Figure 2), several molecular mechanisms of age-associated vascular decline still remained unclear. Based on the literature, the age-related vascular dysfunction seems to be the leading cause for the onset of both cardio- and cerebral vascular diseases. Albeit, the characterization of the molecular mechanisms and of the pathophysiological processes of cerebro- and cardiovascular diseases aging-related have facilitated and improved the pharmacological approaches to several aging diseases in the future researchers should identify and define new genetic polymorphisms able to predict the development of structural and functional alterations of aging such as vascular, diastolic or sympathetic dysfunction aiming to delay aging-related diseases as late as possible promoting a successful life.

**Limitations:** The real limit to the therapeutic intervention on aging is due to the lack in research and detection. The lack in research is directly correlated to all the missing pieces of the aging process from cellular processes such as replication, differentiation, apoptosis to the genetic and biochemical mechanisms. By this we mean that, research in regard to aging is too often projected toward researching diseases correlated with aging and not aging itself. In fact, the second limit is the detection of the aging process, both in its development and in its therapeutic intervention. We have no actual way to evaluate aging, no marker that directly relates to aging. Example of this is the use of anti-oxidant supplementation for aging, of course we have an increased oxidative state in aging and obviously the use of this supplementation betters the oxidative state of the elderly but it does not affect aging itself and even if it did how could we know. These simply means that as of today we have therapies for disease associated with aging but we have no therapy for the mechanism of aging itself and so we can only hope to slow down aging by acting on the outskirts associated with it. As of today, we should follow a double path, one on which we better the therapies associated with aging and one on which we research aging itself and its markers.

In conclusion, we must continue to find the optimal therapies able to slow down aging and its alterations, without neglecting biological studies on large populations to obtain concrete improvements on treatment outcome. In the meanwhile, it is duty of the medical staff to guarantee the best and up-to-date treatment for elderly patients.
